# Supplementary material for: Geographical Authentication of Macrohyporia cocos by a Data Fusion Method Combining Ultra-Fast Liquid Chromatography and Fourier Transform Infrared Spectroscopy
Source: Molecules. 2019 Apr 3;24(7):1320. doi: 10.3390/molecules24071320 (PMC6479993; doi:10.3390/molecules24071320)
Supplement: Supplementary file 1 [file molecules-24-01320-s001.pdf]

Supplementary material

Geographical authentication of *Macrohyporia cocos* by a data fusion method combining ultra-fast liquid chromatography and Fourier transform infrared spectroscopy

Qin-Qin Wang [1,2], Heng-Yu Huang [2,*] and Yuan-Zhong Wang [1,*]

1 Institute of Medicinal Plants, Yunnan Academy of Agricultural Sciences, 650200, Kunming, China; wqq6501@163.com (Q.Q.W.); boletus@126.com (Y.Z.W.)

2 College of Traditional Chinese Medicine, Yunnan University of Traditional Chinese Medicine, 650500, Kunming, China; wqq6501@163.com (Q.Q.W.), hhyhhy96@163.com (H.Y.H.)

* Correspondence: hhyhhy96@163.com (H.Y.H.), boletus@126.com (Y.Z.W.); Tel.: +86-871-6503-3564 (H.Y.H.), +86-871-6503-3575 (Y.Z.W.)

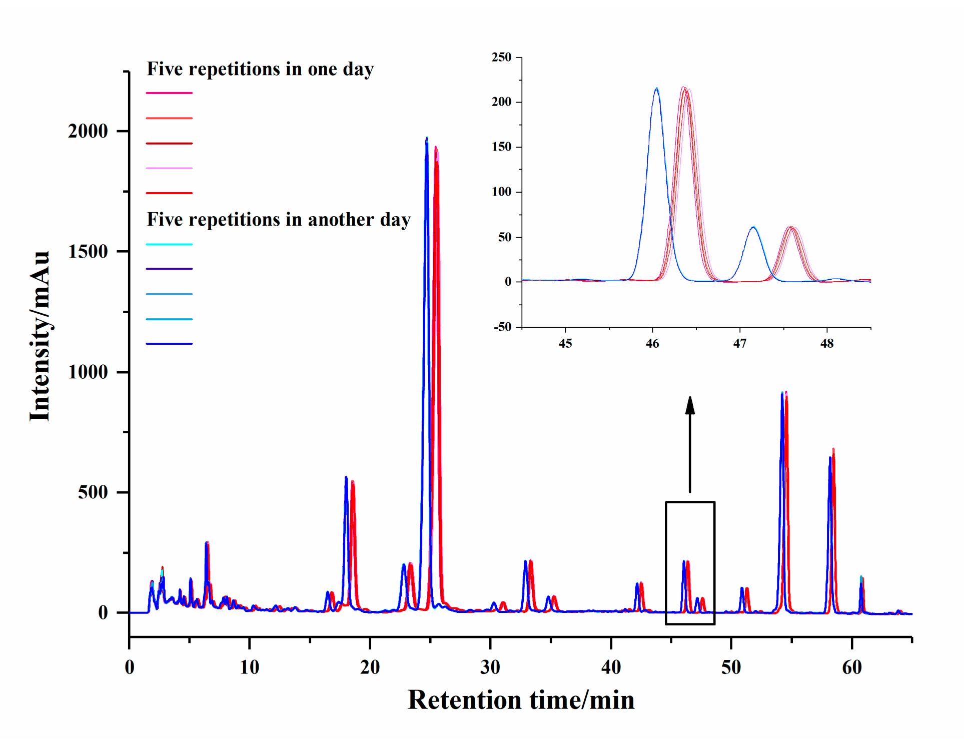

Figure 1. Chromatograms with retention time shifts.

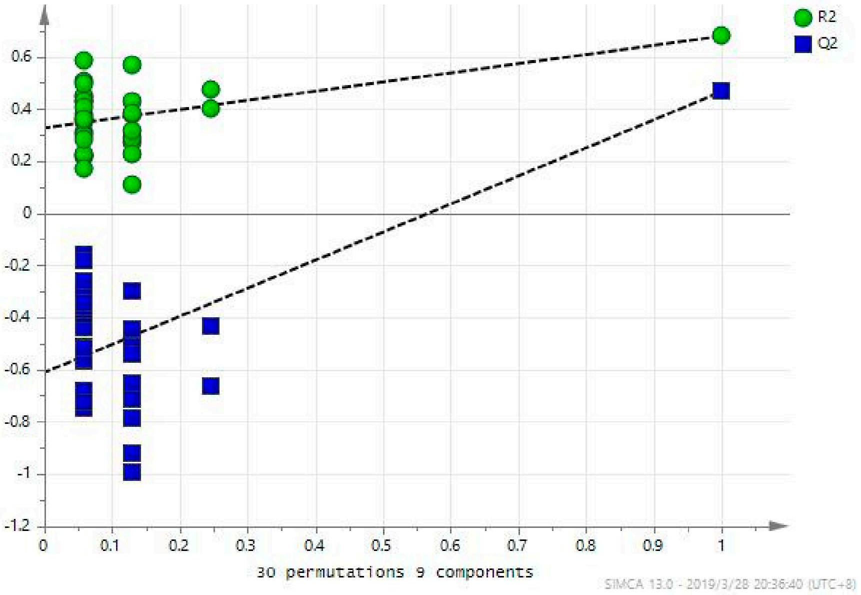

Figure 2. Permutations plot for Poria LC$_{242-210}$ model.

Table 1. Values of RSD in precision, repeatability and stability.

components	intra-day precision (%)			inter-day precisions (%)	repeatability (%)	stability (%)
	first day	second day	third day			
dehydrotumulosic acid	0.9	0.91	0.63	2.81	5.95	0.58
poricoic acid a	0.32	0.57	0.62	2.75	3.51	0.48
dehydropachymic acid	1.24	0.82	0.57	5.68	1.9	0.58
pachymic acid	0.2	0.5	1.01	3.46	2.11	0.71
dehydrotrametenolic acid	1.1	0.46	0.75	5	1.87	0.55

Table 2. The LODs, LOQs, regression equations, correlation coefficients, linear ranges, and recovery rates of five compounds.

components	LOD (μg·mL^{-1})	LOQ (μg·mL^{-1})	regression equation	r^2	linear range (μg·ml^{-1})	recovery rate (%)
dehydrotumulosic acid	0.2	0.5	$Y = 11319140.93X + 74475.78$	0.9983	5.00–999	106.4
dehydropachymic acid	0.24	1.5	$Y = 12988529.78X + 7769.17$	0.9998	2.4–480	98.57
pachymic acid	10.33	103.33	$Y = 7905709.32X + 42996.45$	0.9937	10.3–1240	100.3
dehydrotrametenolic acid	0.49	2	$Y = 21538210.94X + 107194.87$	0.9993	0.49–2450	103.7
poricoic acid a	0.22	0.45	$Y = 19016331.45X + 1507413.80$	0.9973	0.22–6730	96.32
			$Y = 22522136.84X + 42388.59$	0.9999	0.22–1121.95	

Table 3. Comparison of results about data reduction.

data matrix	R^2(cum)	Q^2(cum)	accuracy of calibration set	accuracy of validation set	class	calibration set			validation set		
						sensitivity	specificity	efficiency	sensitivity	specificity	efficiency
LC_{242}	0.6641	0.5287	96.15%	100%	1	0.8571	1.0000	0.9258	1.0000	1.0000	1.0000
					2	1.0000	1.0000	1.0000	1.0000	1.0000	1.0000
					3	1.0000	1.0000	1.0000	1.0000	1.0000	1.0000
					4	1.0000	1.0000	1.0000	1.0000	1.0000	1.0000
					5	1.0000	0.9778	0.9888	1.0000	1.0000	1.0000
					6	1.0000	1.0000	1.0000	1.0000	1.0000	1.0000
					7	0.8571	0.9778	0.9155	1.0000	1.0000	1.0000
					8	1.0000	1.0000	1.0000	1.0000	1.0000	1.0000
LC_{242}-reduing data	0.6634	0.5277	96.15%	100%	1	0.8571	1.0000	0.9258	1.0000	1.0000	1.0000
					2	1.0000	1.0000	1.0000	1.0000	1.0000	1.0000
					3	1.0000	1.0000	1.0000	1.0000	1.0000	1.0000
					4	1.0000	1.0000	1.0000	1.0000	1.0000	1.0000
					5	1.0000	0.9778	0.9888	1.0000	1.0000	1.0000
					6	1.0000	1.0000	1.0000	1.0000	1.0000	1.0000
					7	0.8571	0.9778	0.9155	1.0000	1.0000	1.0000
					8	1.0000	1.0000	1.0000	1.0000	1.0000	1.0000

Table 4. The parameters of each class in PLS-DA model of single techniques.

data matrix		class	calibration set			validation set		
			sensitivity	specificity	efficiency	sensitivity	specificity	efficiency
**Poria**	FTIR	1	1.0000	1.0000	1.0000	1.0000	1.0000	1.0000
		2	1.0000	1.0000	1.0000	0.7500	1.0000	0.8660
		3	1.0000	1.0000	1.0000	1.0000	0.9565	0.9780
		4	1.0000	1.0000	1.0000	1.0000	1.0000	1.0000
		5	1.0000	1.0000	1.0000	1.0000	0.9565	0.9780
		6	1.0000	1.0000	1.0000	1.0000	1.0000	1.0000
		7	1.0000	1.0000	1.0000	1.0000	1.0000	1.0000
		8	1.0000	1.0000	1.0000	0.5000	1.0000	0.7071
	LC_{242}	1	0.8571	1.0000	0.9258	1.0000	1.0000	1.0000
		2	1.0000	1.0000	1.0000	1.0000	1.0000	1.0000
		3	1.0000	1.0000	1.0000	1.0000	1.0000	1.0000
		4	1.0000	1.0000	1.0000	1.0000	1.0000	1.0000
		5	1.0000	0.9778	0.9888	1.0000	1.0000	1.0000
		6	1.0000	1.0000	1.0000	1.0000	1.0000	1.0000
		7	0.8571	0.9778	0.9155	1.0000	1.0000	1.0000
		8	1.0000	1.0000	1.0000	1.0000	1.0000	1.0000
	LC_{210}	1	0.8571	1.0000	0.9258	0.6667	1.0000	0.8165
		2	1.0000	1.0000	1.0000	1.0000	1.0000	1.0000
		3	1.0000	1.0000	1.0000	1.0000	1.0000	1.0000
		4	1.0000	0.9565	0.9780	1.0000	0.7727	0.8790
		5	0.7143	0.9778	0.8357	0.0000	1.0000	0.0000
		6	1.0000	1.0000	1.0000	1.0000	0.9545	0.9770
		7	1.0000	0.9778	0.9888	1.0000	1.0000	1.0000
		8	0.6667	0.9783	0.8076	0.0000	1.0000	0.0000
**Poria Cutis**	FTIR	1	1.0000	1.0000	1.0000	1.0000	1.0000	1.0000
		2	1.0000	1.0000	1.0000	1.0000	1.0000	1.0000
		3	1.0000	1.0000	1.0000	0.6667	1.0000	0.8165
		4	1.0000	1.0000	1.0000	1.0000	1.0000	1.0000
		5	1.0000	1.0000	1.0000	1.0000	0.9565	0.9780
		6	1.0000	1.0000	1.0000	1.0000	1.0000	1.0000
		7	1.0000	1.0000	1.0000	1.0000	1.0000	1.0000
		8	1.0000	1.0000	1.0000	1.0000	1.0000	1.0000
	LC_{242}	1	0.8571	0.9111	0.8837	0.3333	0.8261	0.5247
		2	0.6667	0.9565	0.7985	0.5000	0.9545	0.6908
		3	0.5714	0.9778	0.7475	0.0000	1.0000	0.0000
		4	0.0000	1.0000	0.0000	0.0000	1.0000	0.0000
		5	0.8571	0.9333	0.8944	0.6667	0.8261	0.7421
		6	0.5000	1.0000	0.7071	0.0000	0.9545	0.0000
		7	0.8571	0.8444	0.8508	1.0000	0.7391	0.8597
		8	0.8333	0.9783	0.9029	0.5000	0.9583	0.6922

Table 5. The parameters of each class in PLS-DA model of low-level data fusion.

data matrix		class	calibration set			validation set		
			sensitivity	specificity	efficiency	sensitivity	specificity	efficiency
**Poria**	FTIR-LC_{242}	1	1.0000	1.0000	1.0000	1.0000	1.0000	1.0000
		2	1.0000	1.0000	1.0000	1.0000	1.0000	1.0000
		3	1.0000	1.0000	1.0000	1.0000	1.0000	1.0000
		4	1.0000	1.0000	1.0000	1.0000	1.0000	1.0000
		5	1.0000	1.0000	1.0000	1.0000	1.0000	1.0000
		6	1.0000	1.0000	1.0000	1.0000	1.0000	1.0000
		7	1.0000	1.0000	1.0000	1.0000	1.0000	1.0000
		8	1.0000	1.0000	1.0000	1.0000	1.0000	1.0000
	FTIR-LC_{210}	1	1.0000	1.0000	1.0000	1.0000	1.0000	1.0000
		2	1.0000	1.0000	1.0000	1.0000	1.0000	1.0000
		3	1.0000	1.0000	1.0000	1.0000	1.0000	1.0000
		4	1.0000	1.0000	1.0000	1.0000	1.0000	1.0000
		5	1.0000	1.0000	1.0000	1.0000	1.0000	1.0000
		6	1.0000	1.0000	1.0000	1.0000	1.0000	1.0000
		7	1.0000	1.0000	1.0000	1.0000	1.0000	1.0000
		8	1.0000	1.0000	1.0000	1.0000	1.0000	1.0000
	$LC_{242-210}$	1	1.0000	1.0000	1.0000	1.0000	1.0000	1.0000
		2	1.0000	1.0000	1.0000	1.0000	1.0000	1.0000
		3	1.0000	1.0000	1.0000	1.0000	1.0000	1.0000
		4	1.0000	0.9783	0.9891	1.0000	0.9091	0.9535
		5	0.8571	1.0000	0.9258	0.3333	1.0000	0.5774
		6	1.0000	1.0000	1.0000	1.0000	1.0000	1.0000
		7	1.0000	1.0000	1.0000	1.0000	1.0000	1.0000
		8	1.0000	1.0000	1.0000	1.0000	1.0000	1.0000
	FTIR-$LC_{242-210}$	1	1.0000	1.0000	1.0000	1.0000	1.0000	1.0000
		2	1.0000	1.0000	1.0000	1.0000	1.0000	1.0000
		3	1.0000	1.0000	1.0000	1.0000	1.0000	1.0000
		4	1.0000	1.0000	1.0000	1.0000	1.0000	1.0000
		5	1.0000	1.0000	1.0000	1.0000	1.0000	1.0000
		6	1.0000	1.0000	1.0000	1.0000	1.0000	1.0000
		7	1.0000	1.0000	1.0000	1.0000	1.0000	1.0000
		8	1.0000	1.0000	1.0000	1.0000	1.0000	1.0000
**Poria Cutis**	FTIR-LC_{242}	1	1.0000	1.0000	1.0000	1.0000	1.0000	1.0000
		2	1.0000	1.0000	1.0000	1.0000	1.0000	1.0000
		3	1.0000	1.0000	1.0000	1.0000	1.0000	1.0000
		4	1.0000	1.0000	1.0000	1.0000	1.0000	1.0000
		5	1.0000	1.0000	1.0000	1.0000	1.0000	1.0000
		6	1.0000	1.0000	1.0000	1.0000	1.0000	1.0000
		7	1.0000	1.0000	1.0000	1.0000	1.0000	1.0000
		8	1.0000	1.0000	1.0000	1.0000	1.0000	1.0000
	FTIR-$LC_{242-210}$	1	1.0000	1.0000	1.0000	1.0000	1.0000	1.0000
		2	1.0000	1.0000	1.0000	1.0000	1.0000	1.0000
		3	1.0000	1.0000	1.0000	1.0000	1.0000	1.0000
		4	1.0000	1.0000	1.0000	1.0000	1.0000	1.0000
		5	1.0000	1.0000	1.0000	1.0000	1.0000	1.0000
		6	1.0000	1.0000	1.0000	1.0000	1.0000	1.0000
		7	1.0000	1.0000	1.0000	1.0000	1.0000	1.0000
		8	1.0000	1.0000	1.0000	1.0000	1.0000	1.0000

**combination data of two medicinal parts**	FTIR	1	1.0000	1.0000	1.0000	1.0000	1.0000	1.0000
		2	1.0000	1.0000	1.0000	1.0000	1.0000	1.0000
		3	1.0000	1.0000	1.0000	1.0000	1.0000	1.0000
		4	1.0000	1.0000	1.0000	1.0000	1.0000	1.0000
		5	1.0000	1.0000	1.0000	1.0000	1.0000	1.0000
		6	1.0000	1.0000	1.0000	1.0000	1.0000	1.0000
		7	1.0000	1.0000	1.0000	1.0000	1.0000	1.0000
		8	1.0000	1.0000	1.0000	1.0000	1.0000	1.0000
	LC_{242}	1	1.0000	1.0000	1.0000	1.0000	1.0000	1.0000
		2	1.0000	1.0000	1.0000	1.0000	1.0000	1.0000
		3	1.0000	1.0000	1.0000	1.0000	1.0000	1.0000
		4	1.0000	1.0000	1.0000	1.0000	1.0000	1.0000
		5	1.0000	1.0000	1.0000	1.0000	1.0000	1.0000
		6	1.0000	1.0000	1.0000	1.0000	1.0000	1.0000
		7	1.0000	1.0000	1.0000	1.0000	1.0000	1.0000
		8	1.0000	1.0000	1.0000	1.0000	1.0000	1.0000
	LC_{210}	1	1.0000	1.0000	1.0000	1.0000	1.0000	1.0000
		2	1.0000	0.9783	0.9891	1.0000	1.0000	1.0000
		3	1.0000	1.0000	1.0000	1.0000	1.0000	1.0000
		4	1.0000	0.9565	0.9780	1.0000	1.0000	1.0000
		5	0.7143	1.0000	0.8452	0.0000	1.0000	0.0000
		6	1.0000	1.0000	1.0000	1.0000	1.0000	1.0000
		7	1.0000	1.0000	1.0000	1.0000	1.0000	1.0000
		8	0.8333	1.0000	0.9129	1.0000	0.8750	0.9354

Table 6. The parameters of each class in PLS-DA model of mid-level data fusion.

data matrix		class	calibration set			validation set		
			sensitivity	specificity	efficiency	sensitivity	specificity	efficiency
**Poria**	FTIR-LC_{242}	1	1.0000	1.0000	1.0000	1.0000	1.0000	1.0000
		2	1.0000	1.0000	1.0000	1.0000	1.0000	1.0000
		3	1.0000	1.0000	1.0000	1.0000	1.0000	1.0000
		4	1.0000	1.0000	1.0000	1.0000	1.0000	1.0000
		5	1.0000	1.0000	1.0000	1.0000	1.0000	1.0000
		6	1.0000	1.0000	1.0000	1.0000	1.0000	1.0000
		7	1.0000	1.0000	1.0000	1.0000	1.0000	1.0000
		8	1.0000	1.0000	1.0000	1.0000	1.0000	1.0000
	FTIR-LC_{210}	1	1.0000	0.9778	0.9888	1.0000	1.0000	1.0000
		2	0.8333	1.0000	0.9129	1.0000	1.0000	1.0000
		3	1.0000	1.0000	1.0000	1.0000	1.0000	1.0000
		4	1.0000	0.9783	0.9891	1.0000	1.0000	1.0000
		5	1.0000	1.0000	1.0000	0.6667	1.0000	0.8165
		6	1.0000	1.0000	1.0000	1.0000	1.0000	1.0000
		7	1.0000	1.0000	1.0000	1.0000	1.0000	1.0000
		8	0.8333	1.0000	0.9129	1.0000	0.9583	0.9789
	FTIR-$LC_{242\text{-}210}$	1	1.0000	1.0000	1.0000	1.0000	1.0000	1.0000
		2	1.0000	1.0000	1.0000	1.0000	1.0000	1.0000
		3	1.0000	1.0000	1.0000	1.0000	1.0000	1.0000
		4	1.0000	1.0000	1.0000	1.0000	1.0000	1.0000
		5	1.0000	1.0000	1.0000	1.0000	1.0000	1.0000
		6	1.0000	1.0000	1.0000	1.0000	1.0000	1.0000
		7	1.0000	1.0000	1.0000	1.0000	1.0000	1.0000
		8	1.0000	1.0000	1.0000	1.0000	1.0000	1.0000
**Poria Cutis**	FTIR-LC_{242}	1	1.0000	1.0000	1.0000	1.0000	1.0000	1.0000
		2	1.0000	1.0000	1.0000	0.5000	1.0000	0.7071
		3	1.0000	1.0000	1.0000	1.0000	1.0000	1.0000
		4	1.0000	1.0000	1.0000	1.0000	1.0000	1.0000
		5	1.0000	1.0000	1.0000	1.0000	1.0000	1.0000
		6	1.0000	1.0000	1.0000	1.0000	1.0000	1.0000
		7	1.0000	1.0000	1.0000	1.0000	1.0000	1.0000
		8	1.0000	1.0000	1.0000	1.0000	0.9167	0.9574
	FTIR-LC_{210}	1	1.0000	1.0000	1.0000	1.0000	1.0000	1.0000
		2	1.0000	1.0000	1.0000	0.7500	1.0000	0.8660
		3	1.0000	1.0000	1.0000	1.0000	1.0000	1.0000
		4	1.0000	1.0000	1.0000	1.0000	1.0000	1.0000
		5	1.0000	1.0000	1.0000	1.0000	1.0000	1.0000
		6	1.0000	1.0000	1.0000	1.0000	1.0000	1.0000
		7	1.0000	1.0000	1.0000	1.0000	1.0000	1.0000
		8	1.0000	1.0000	1.0000	1.0000	0.9583	0.9789
	$LC_{242\text{-}210}$	1	1.0000	1.0000	1.0000	1.0000	1.0000	1.0000
		2	0.8333	1.0000	0.9129	0.7500	0.9545	0.8461
		3	1.0000	0.9778	0.9888	1.0000	0.9565	0.9780
		4	1.0000	0.9565	0.9780	1.0000	1.0000	1.0000
		5	1.0000	0.9111	0.9545	0.3333	0.9565	0.5647
		6	1.0000	0.8913	0.9441	1.0000	0.9091	0.9535
		7	0.0000	1.0000	0.0000	0.0000	1.0000	0.0000
		8	0.3333	1.0000	0.5774	0.5000	0.9167	0.6770

	FTIR-$LC_{242-210}$	1	1.0000	1.0000	1.0000	1.0000	1.0000	1.0000
		2	1.0000	1.0000	1.0000	0.7500	1.0000	0.8660
		3	1.0000	1.0000	1.0000	1.0000	1.0000	1.0000
		4	1.0000	1.0000	1.0000	1.0000	1.0000	1.0000
		5	1.0000	1.0000	1.0000	1.0000	1.0000	1.0000
		6	1.0000	1.0000	1.0000	1.0000	1.0000	1.0000
		7	1.0000	1.0000	1.0000	1.0000	0.9565	0.9780
		8	1.0000	1.0000	1.0000	1.0000	1.0000	1.0000
**combination data of two medicinal parts**	FTIR	1	1.0000	1.0000	1.0000	1.0000	1.0000	1.0000
		2	0.8333	1.0000	0.9129	0.2500	1.0000	0.5000
		3	1.0000	0.9778	0.9888	1.0000	1.0000	1.0000
		4	1.0000	1.0000	1.0000	1.0000	1.0000	1.0000
		5	1.0000	1.0000	1.0000	1.0000	1.0000	1.0000
		6	1.0000	1.0000	1.0000	1.0000	1.0000	1.0000
		7	1.0000	1.0000	1.0000	1.0000	1.0000	1.0000
		8	1.0000	1.0000	1.0000	1.0000	0.8750	0.9354
	LC_{242}	1	1.0000	1.0000	1.0000	1.0000	1.0000	1.0000
		2	1.0000	1.0000	1.0000	1.0000	1.0000	1.0000
		3	1.0000	1.0000	1.0000	1.0000	1.0000	1.0000
		4	1.0000	0.9783	0.9891	1.0000	1.0000	1.0000
		5	0.8571	1.0000	0.9258	1.0000	1.0000	1.0000
		6	1.0000	1.0000	1.0000	1.0000	1.0000	1.0000
		7	1.0000	1.0000	1.0000	1.0000	1.0000	1.0000
		8	1.0000	1.0000	1.0000	1.0000	1.0000	1.0000
	LC_{210}	1	1.0000	1.0000	1.0000	1.0000	1.0000	1.0000
		2	1.0000	1.0000	1.0000	0.7500	1.0000	0.8660
		3	1.0000	1.0000	1.0000	1.0000	1.0000	1.0000
		4	1.0000	0.9565	0.9780	1.0000	0.9545	0.9770
		5	0.8571	1.0000	0.9258	0.3333	1.0000	0.5774
		6	1.0000	1.0000	1.0000	1.0000	1.0000	1.0000
		7	1.0000	1.0000	1.0000	1.0000	0.9130	0.9555
		8	0.8333	1.0000	0.9129	1.0000	1.0000	1.0000

Table 7. The results of feature extraction on PCA.

data matrix	optimal number of PCs	R^2(cum)
**Poria**		
FTIR	9	0.7329
LC_{242}	23	0.9739
LC_{210}	13	0.9092
**Poria Cutis**		
FTIR	10	0.7454
LC_{242}	10	0.9427
LC_{210}	17	0.9581
